# Severity of erectile dysfunction is highly correlated with the syntax score in patients undergoing coronariography

**DOI:** 10.1590/S1677-5538.IBJU.2015.0002

**Published:** 2016

**Authors:** Weslley Santiago Andrade, Paulo Oliveira, Humberto Laydner, Eduardo Jose Pereira Ferreira, Jose Augusto Soares Barreto

**Affiliations:** 1 Universidade Federal de Sergipe, Aracaju, Sergipe, Brazil,; 2 Universidade Estadual de Feira de Santana, Feira de Santana, Bahia, Brazil,; 3 University Hospitals-Urology, Cleveland, OH, USA

**Keywords:** Erectile Dysfunction, Coronary Angiography, Patients

## Abstract

**Objective:**

To investigate the association between the severity of erectile dysfunction (ED) and coronary artery disease (CAD) in men undergoing coronary angiography for angina or acute myocardial infarct (AMI).

**Material and Methods:**

We studied 132 males who underwent coronary angiography for first time between January and November 2010. ED severity was assessed by the international index of erectile function (IIEF-5) and CAD severity was assessed by the Syntax score. Patients with CAD (cases) and without CAD (controls) had their IIEF-5 compared. In the group with CAD, their IIEF-5 scores were compared to their Syntax score results.

**Results:**

We identified 86 patients with and 46 without CAD. The IIEF-5 score of the group without CAD (22.6±0.8) was significantly higher than the group with CAD (12.5±0.5; p<0.0001). In patients without ED, the Syntax score average was 6.3±3.5, while those with moderate or severe ED had a mean Syntax score of 39.0±11.1. After adjustment, ED was independently associated to CAD, with an odds ratio of 40.6 (CI 95%, 14.3-115.3, p<0.0001). The accuracy of the logistic model to correctly identify presence or absence of CAD was 87%, with 92% sensitivity and 78% specificity. The average time that ED was present in patients with CAD was 38.8±2.3 months before coronary symptoms, about twice as high as patients without CAD (18.0±5.1 months).

**Conclusions:**

ED severity is strongly and independently correlated with CAD complexity, as assessed by the Syntax score in patients undergoing coronariography for evaluation of new onset coronary symptoms.

## INTRODUCTION

Atherosclerosis is a multifactorial chronic inflammatory disease which occurs as a response to endothelial damage, affecting mostly the intimal layer of multiple diameter arteries. In at least half of the patients, the first presentation of atherosclerotic disease is an acute coronary event. In 68% of sudden deaths, patients did not have previous symptoms of coronary heart disease (1). There is a huge effort worldwide for early detection of coronary artery disease (CAD), due to its elevated morbidity and mortality rates as well as significant social-economics consequences. It is estimated that atherothrombotic events will continue to be the leading cause of death as far as 2020 (2).

Although invasive and requiring highly specialized physics, equipment and personnel structure, the gold standard test to determine the presence and severity of CAD is coronariography. The Syntax score developed for the SYNTAX trial (Synergy Between PCI with Taxus and Cardiac Surgery) presents a thorough way to analyze the severity of CAD (3, 4).

Erectile dysfunction (ED) is the inability to achieve and maintain erection for satisfactory sexual intercourse (5) and its global prevalence ranges from 2 to 86% (6). ED has risk factors and physiopathology basis similar to those of CAD, with endothelial dysfunction as a common denominator affecting several vascular beds of multiple diameters (7). ED can be easily assessed by using the abridged International Index of Erectile Function (IIEF-5) questionnaire (8). Besides affecting sexuality, ED has been increasingly recognized for its ability to detect insidious CAD (9, 10).

There is no non-invasive method, clinical sign or laboratory test capable of detecting all individuals who will develop CAD. ED may be an early clinical marker of CAD with association to the degree of ED and the severity of coronary artery disease in a large portion of men. Herein, we evaluated the association between the complexity of CAD measured by the Syntax score and the severity of ED measured by the IIEF-5 in men who underwent coronary angiography.

## MATERIAL AND METHODS

Male patients undergoing coronary angiography for the first time between January and November 2010 were included in this case-control study. During this period, 1773 patients underwent coronary angiography. Based on convenience sampling, 132 patients consecutively submitted to first coronary angiography for diagnosis of CAD were selected according to the following criteria: age between 40 and 70 years old, hemodynamic stability, full recovery from the procedure with consciousness and orientation (average recovery time of 3 hours after the procedure), at least 2 risk factors for CAD [hypertension, dyslipidemia, hyperglycemia, smoking, family history of stroke, or acute myocardial infarction (AMI)], absence of previous cardiovascular events (first event).

Exclusion criteria were: ED after radical prostatectomy, ED secondary to a neurological lesion, refusal to participate in the study. Five patients were excluded because they were not fully recovered from the procedure.

Patients were clinically evaluated and filled both a demographic and the IIEF-5 questionnaires. The recorded coronariography films were analyzed with the Syntax Score to evaluate the complexity of the coronary lesions by an experienced cardiologist who was blind to the patient’s erectile function status. The SYNTAX score is the sum of the points assigned to each individual lesion with >50% diameter narrowing in vessels >1.5mm diameter in the coronary tree. A computer algorithm is then queried and a summed value is generated.

Patients were divided into two groups according to the exam results: Group-1 (test) had obstruction equal to or greater than 50% of the vessel lumen and Group-2 (control) without CAD detected on coronariography. Patients on Group-2 should have at least one additional exam, such as stress test, stress echocardiography, or myocardial scintigraphy to rule out CAD.

Group-2 patients were further classified according to their indication for the exam in acute (unstable angina, AMI) or chronic (stable angina) cases. The duration of ED was considered in relation to the first coronary symptom.

The IIEF-5 questionnaire was given after the patient fully recovered from the angiographic procedure. The IIEF-5 ranges from 5 to 25 points, classifying ED into one of five possible categories: severe (5-7 points), moderate (8-11), mild to moderate (12-16), mild (17-21), and absence of ED (22-25). The IIEF-5 questions refer to the patient’s symptoms in the previous 6 months and not only to the moment when they are answering the questions.

This study was approved by the ethics committee of our Institution under the protocol number 1274.0.000.107-09. Patients were included in the study only after informed consent was obtained.

### Statistical analysis

The continuous and categorical variables were described as averages with standard deviation and frequencies with 95% confidence interval, respectively. The Shapiro-Wilk test was used to evaluate the assumption of normality. We used either the Pearson Chi-square or the Fisher’s exact test when appropriate to test the hypothesis relative to categorical variables. The Student’s t-test for independent samples was used for the comparison between groups with CAD and without CAD. The comparison of the variable “SYNTAX score” between the groups with different degrees of ED was performed with analysis of variance (ANOVA) followed by the Tukey post-hoc. The comparison of the variable “IIEF-5” between the test and control groups was performed with a general linear model with a single factor (group) adjusted for the variables “age” and “use of diuretics”. The forward stepwise logistic regression method was used to evaluate ED as a predictor of CAD. The ability of the model to discriminate patients with CAD and without CAD with the receiver operating characteristic (ROC) curve was also analyzed. The ability of the ROC curve to discriminate between cases with CAD and without CAD for the variable “IIEF-5” was also evaluated. The p value of 0.05 was considered significant. The SPSS software version 18.0® was used for statistical analysis.

## RESULTS

**Figure 1:**
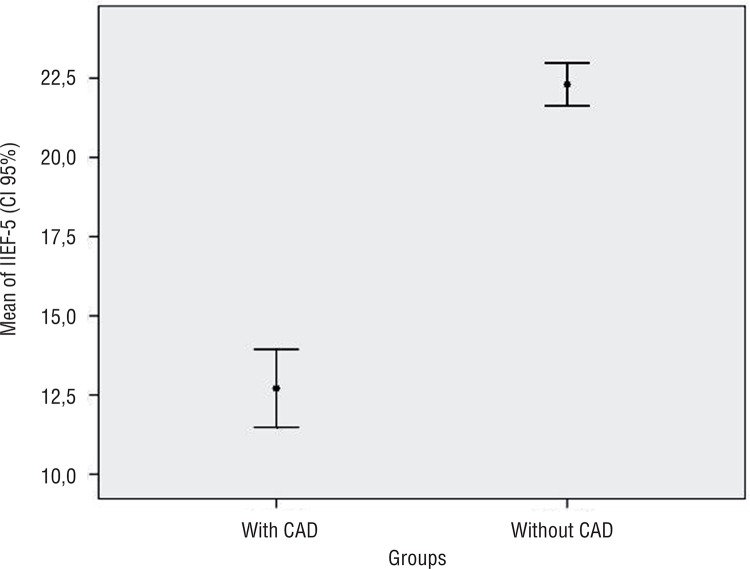
IIEF-5 versus Coronary Artery Disease

We evaluated 132 male patients, 86 with CAD and 46 without CAD. Patient’s characteristics are shown in Table-1. The mean age was 58.6±8.4 years. Hypertension and diabetes were present in 95.4% and 31% of the patients, respectively. The test and control groups were significantly different regarding mean age, use of diuretics, and IIEF-5 (p=0.02, p<0.0001, and p<0.0001, respectively). The IIEF-5 score of the group without CAD (adjusted mean 22.6±0.8) was significantly higher than the score of the group with CAD (adjusted mean 12.5±0.5) after adjustment for age and use of diuretics, with an average difference of 10.1±0.9 (IC 95%, 8.2–12.0, p<0.0001) between the groups (Figure-1). ED was present in 89 patients (67.4%), 46% of which were moderate or severe (Table-2).

**Table 1:** Baseline characteristics.

	Group 1 (n=86) (with CAD)	Group 2 (n=46) (without CAD)	P value
Age	59.8±8.2	56.3±8.8	= 0.02
Sistolic BP	156.5±19.0	154.7±17.4	0.6
Diastolic BP	92.7±9.4	92.5±9.0	0.9
BMI	27.2±3.1	27.1±4.2	0.9
AC	103.0±20.1	99.0±10.,8	0.2
Glucose	107.9±39.8	107.9±39.7	0.9
Total cholesterol	214.3±48.6	199.4±72.4	0.2
HDL	39.5±7.4	41.0±9.6	0.2
LDL	139.0±42.8	125.8±64.0	0.2
Triglycerids	178.7±68.3	160.1±69.2	0.2
Total IIEF-5	12.7±5.7	22.03±2.3	< 0.0001
Smoking	48 (55.8%)	21 (45.7%)	0.3
Family history of stroke	28 (32.6%)	12 (26.1%)	0.6
Family history of CVD	30 (34.9%)	15 (32.6%)	0.8
Hypertension	82 (95.3%)	44 (95.7%)	0.9
Diabetes Mellitus	29 (33.7%)	12 (26.1%)	0.4
**ETHNICITY**			
White	66 (76.74%)	32 (69.57%)	0.4
No-White	14(23.26%)	20 (30.43%)	0.4
**MEDICATIONS IN USE**			
Diuretics	15 (17.4 %)	25 (54.3%)	< 0.0001
Adrenergic inhibitors	44 (51.2 %)	21 (45.7%)	0.6
Vasodilators	18 (20.9%)	07 (15.2%)	0.5
CCB	09 (10.5%)	08 (17.4%)	0.3
ACE Inhibitors	31 (36.0%)	18 (39.1%)	0.8
ARB	13 (15.1%)	11 (23.9%)	0.2

**HDL =** high density lipoprotein; **LDL =** low density lipoprotein; **CVD =** cardiovascular disease; **BMI =** body mass index; **AC =** abdominal circunference; **CCB =** calcium channel blockers; **ACE =** angiotensin-converting enzyme; **ARB =** angiotensina II receptor blockers

**Table 2:** – Erectile dysfunction severity (IIEF-5).

Erectyle dysfunction severity	n (%)	CI 95%
No ED	43 (32.6 %)	25.0 – 40.2
Mild ED	28 (21.2%)	14.4 – 28.0
Mild to moderate ED	20 (15.2%)	9.1 – 21.2
Moderate ED	19 (14.4%)	8.3 – 20.5
Severe ED	22 (16.7%)	10.6 – 23.5

**Figure 2:**
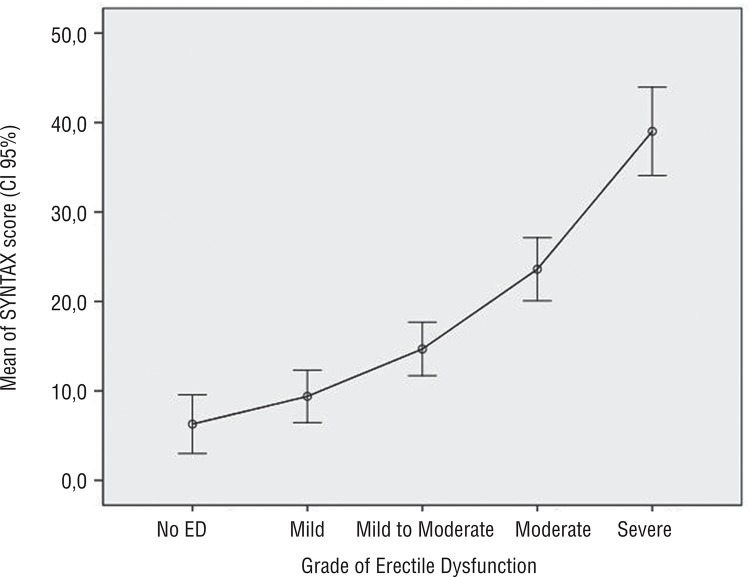
Grade of ED according to Syntax Score

In the 86 patients with CAD, the Syntax score averages increased exponentially with increasing severity of ED. In patients with normal erectile function, the Syntax score average was 6.3±3.5, while those patients with moderate or severe ED had a mean Syntax score of 39.0±11.1 (Figure-2).

After adjustment for age, total cholesterol, LDL cholesterol, and triglycerides, ED was the only variable independently associated to CAD, with odds ratio of 40.6 (CI 95%, 14.3–115.3, p<0.0001). The accuracy of the logistic model to correctly identify the presence or absence of CAD was 87.1% after adjustment, with sensibility of 91.9% and specificity of 78.3% for diagnosis of CAD. The area under the ROC curve was 0.851±0.040 (p<0.001, CI 95%, 0.773–0.929), demonstrating a good discriminant ability (Table-3).

**Table 3:** Factors associated with CAD.

Variable	Odds ratio (adjusted)	CI 95%	P
Age	-	-	0.55
Total cholesterol	-	-	0.83
LDL	-	-	0.98
Triglycerids	-	-	0.82
Smoking	-	-	0.78
Erectile Dysfunction	40.6	14.3 -115.3	<0.0001

**Figure 3:**
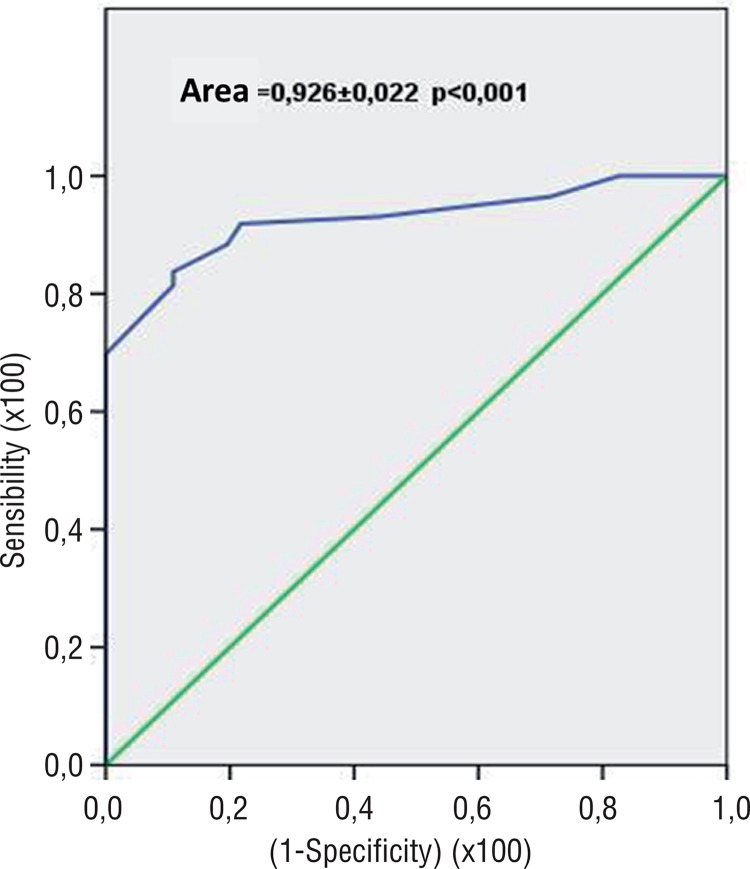
ROC curve of IIEF-5 and CAD

The IIEF-5 questionnaire had an exceptional discriminant ability for presence or absence of CAD (area under the ROC curve 0.922±0.022, p<0.001), with sensibility of 91.2%, specificity of 78.3%, and an IIEF-5 cutoff value of 21.5 (Figure-3).

The average time that ED was present in patients with CAD was 38.8±2.3 months before the coronariography. The average time of ED in patients with acute disease was 38.4±19.2 months (Table-4).

**Table 4:** – Time with Erectile Dysfunction (n=89).

Groups	Time (months)	CI 95%	P
Without DAC	18.0±5.1	7.9–28.1	<0.0001
With DAC	38.8±2.3	34.4–43.3	
Acute cases*	38.4±19.2		0.76
Chronic cases**	39.9±22.9		

* First acute myocardial infarction (AMI) episode, AMI without ST segment elevation or unstable angina; **Stable Angina or thoracic discomfort for > 2 months

## DISCUSSION

In our study, the IIEF-5 questionnaire had exceptional discriminant ability for the diagnosis of CAD, when compared with the Syntax score. We identified a clear correlation between the severity of ED and the complexity of CAD, which has been previously observed in the literature (11-13). However, the method that we used to evaluate the complexity of coronary disease differed from those used by other authors. Greenstein et al. used the number of vessels with at least one significant lesion. They found that patients with a single vessel affected had erections more frequently, more rigid, and more easily to achieve than patients with 2 or 3 vessels affected (11). Montorsi et al. used the Gensini’s score, which estimates the amount of myocardium affected by each coronary lesion. They identified a significant increase of the Gensini’s score proportional to the decrease of the IIEF-5 score (14).

To our knowledge, this is the first study to use the Syntax score as the instrument to evaluate the complexity of coronary lesions in patients with ED. The Syntax was developed as a comprehensive angiographic scoring system aiming to assist in patient selection and risk stratification of patients with extensive coronary artery disease undergoing revascularization with either percutaneous coronary intervention (PCI) or coronary-artery bypass grafting (CAPG) (15). According to the characteristics of the lesions, the SYNTAX score is able to identify patients who are good candidates for PCI, classifying them into low risk (0-22 points), intermediate risk (23-32 points), and high risk (≥33 points) patients. It is also a good predictor of adverse events in patients with multiarterial coronary disease and/or with lesions in the left coronary artery main trunk who underwent PCI. Hence, this instrument has a good discriminatory power for risk assessment (16).

The highly significant correlation that we found between an increased Syntax score and a decreased IIEF-5 score suggests that the ED severity may be an important factor to be assessed before the indication of myocardial revascularization, either by PCI or CABG, which needs to be confirmed in a study designed for this purpose.

The IIEF-5 is widely used because of its easy application and reproducibility. It has a sensibility of 98% and specificity of 88% (8, 17).

In our study, we did not find any significant difference in the presence of ED between acute and chronic cases. Montorsi et al. found a different prevalence of ED between patients with acute and chronic CAD, with a lower ED rate in acute cases (14).

The fact that we did not reproduce this finding could be possibly explained by the limited number of chronic cases in our study.

In our study, the mean time interval between the onset of ED and coronary symptom was 38.8 months in the group with CAD, more than two times greater than in the control group (18 months). Montorsi et al. found similar numbers in a previous study with 300 men with acute chest pain in whom ED symptoms became clinically evident prior to CAD symptoms in 67% of the patients. They also observed a mean time interval between the onset of ED and CAD of 38.8 months (18).

There is evidence of association between penile vascular alterations and abnormalities in the stress test of asymptomatic individuals (19-21). For this reason, all patients included in the control group of our study were required to have, besides the coronariography, at least one exam (stress test, stress echocardiography, or myocardial scintigraphy) to rule out ischemic heart disease.

ED and CAD share common risk and etiology factors, with atherosclerosis and endothelial dysfunction promoting vascular insufficiency (22). ED is as a highly sensitive, specific, and accurate marker for potential cardiovascular events. A meta-analysis of 7 cohort studies with 45.558 patients showed an adjusted relative risk of 1.47 (95% CI, 1.29-1.66, P<0.001) for CVD events in patients with ED in comparison with healthy subjects (23).

Age and use of diuretics were the only ED risk factors that differed significantly between the groups. Both are well known risk factors for ED (24, 25). However, the difference of the average IIEF-5 scores between the groups with and without CAD persisted significant even after adjustment for those variables. Riedner et al. observed that men younger than 60 years old with ED are at increased risk for more severe CAD, independently of risk factors for ED and CAD (26).

Limitations of this study include its retrospective nature, which inherently has the potential for selection bias, lack of penile Doppler ultrasound or testosterone levels. Also, the study was conducted in a specific population who carried, at least, two risk factors for atherosclerosis and had an indication for coronary angiography for a clinical suspicion. Hence, its results should be further verified for external validation. Nevertheless, our results associated to the substantial evidence in the literature strongly suggests that the simple assessment of ED severity with a validated tool should not be neglected in the daily practice, as it may aid in the early diagnosis of CAD and prevention of morbidity and mortality associated to coronary disease. The IIEF-5 is an inexpensive and easily applicable tool that may aid in the decision to perform screening exams for CAD in men without coronary symptoms. This holds true especially for patients with other risk factors for CAD. Application of this tool is particularly useful in the daily practice, where methods to detect latent cardiovascular events are extremely important. Health professionals in general should consider ED as a significant medical condition and run a thorough clinical evaluation looking for cardiovascular risk factors. The time when patients first complain of ED may represent a timely window for the early diagnosis and treatment of cardiovascular disease.

## CONCLUSIONS

The severity of ED as assessed by the IIEF-5 is strongly and independently correlated with the Syntax score in patients undergoing coronariography for evaluation of new onset coronary symptoms. This study further confirms the importance of ED and the IIEF-5 questionnaire for the early identification of patients at increased risk for CAD, which could elicit measures to prevent major cardiovascular events in this population. More studies are needed to determine whether application of the IIEF-5 could also be extended to a general population without other CAD risk factors.
